# Prevalence and Correlates of HIV Testing among Young People Enrolled in Non-Formal Education Centers in Urban Chiang Mai, Thailand: A Cross-Sectional Study

**DOI:** 10.1371/journal.pone.0153452

**Published:** 2016-04-12

**Authors:** Patou Masika Musumari, Arunrat Tangmunkongvorakul, Kriengkrai Srithanaviboonchai, Sawang Yungyuankul, Teeranee Techasrivichien, S. Pilar Suguimoto, Masako Ono-Kihara, Masahiro Kihara, Suwat Chariyalertsak

**Affiliations:** 1 Department of Global Health and Socio-epidemiology, Kyoto University School of Public Health, Kyoto, Japan; 2 Research Institute for Health Sciences, Chiang Mai University, Chiang Mai, Thailand; 3 Faculty of Medicine, Chiang Mai University, Chiang Mai, Thailand; 4 Center of Medical Education, Graduate School of Medicine, Kyoto University, Kyoto, Japan; University of Toronto, CANADA

## Abstract

**Background:**

HIV testing is the gateway to HIV prevention, treatment, and care. Despite the established vulnerability of young Thai people to HIV infection, studies examining the prevalence and correlates of HIV testing among the general population of Thai youth are still very limited. This study investigates socio-demographic, behavioral, and psychosocial factors associated with HIV testing among young Thai people enrolled in Non-formal Education Centers (NFEC) in urban Chiang Mai, Northern Thailand.

**Methods:**

This was a cross-sectional quantitative study conducted among young unmarried Thai youth—between the ages of 15 and 24—who were enrolled in NFEC in urban Chiang Mai. Multiple logistic regressions were used to identify correlates of “ever tested for HIV” among the sexually active participants.

**Findings:**

Of the 295 sexually active participants, 27.3% reported “ever tested for HIV;” 65.4% “did not consistently use condom;” and 61.7% “had at least 2 lifetime partners.” We found that “self-efficacy” (AOR, 4.92; CI, 1.22–19.73); “perception that it is easy to find a location nearby to test for HIV” (AOR, 4.67; CI, 1.21–18.06); “having at least 2 lifetime sexual partners” (AOR, 2.05; CI, 1.09–3.85); and “ever been pregnant or made someone pregnant” (AOR, 4.06; CI, 2.69–9.15); were associated with increased odds of having ever been tested. On the other hand, “fear of HIV test results” (AOR, 0.21; CI, 0.08–0.57) was associated with lower odds of ever having been tested for HIV.

**Conclusion:**

The main finding is that a substantially high proportion of Thai youth is engaged in risky sexual behaviors—yet reports low rates of ever having been tested for HIV. This highlights an urgent need to develop appropriate interventions—based on the identified correlates of HIV testing. There is also an urgent need to enhance HIV testing and to promote safer sexual behaviors among young Thai people—particularly those who are out-of-school.

## Introduction

The prospect of ending the HIV/AIDS epidemic has crystalized over time. Increasing evidence today shows the potential for antiretroviral therapy (ART). It has a strong therapeutic effect, and it also has the ability to dramatically reduce both the transmission and acquisition of HIV infection [1]. HIV testing—as the gateway to effective HIV prevention, treatment, and care—must absolutely be optimized, before this enthusiastic hope to end the HIV/AIDS epidemic could translate to palpable reality. There is extensive literature in support of the finding that individuals substantially reduce risky sexual behaviors after they become aware of their HIV status [2, 3]. Moreover—for infected individuals—HIV testing prompts early initiation of ART, which, in turn, is associated with significantly reduced risk of HIV transmission [1].

However, today, the proportion of individuals who know their HIV status still remains unacceptably low. At the global level, as of the end of 2012, only 50% of people were aware of their HIV status, with great variation within and between countries and regions [4]. This sobering fact indicates that—despite the current expansion of HIV prevention, treatment and care programs worldwide—tremendous efforts are still necessary to improve HIV testing behavior, in order to achieve the optimum possible level of prevention and treatment of HIV infection.

In Thailand, the first anonymous HIV testing and counseling (HTC) clinic was established by the Thai Red Cross Society in mid-1991. The Ministry of Public Health (MoPH) aligned its efforts with those of the Thai Red Cross Society. It adopted HIV counseling and testing (HTC) as part of the country’s prevention strategies in 1992, and subsequently made the service available in all government health clinics in all the provinces [5, 6]. Currently, HIV testing services are delivered through public health facilities, such as government hospitals; government clinics; and health centers. Testing services are also delivered at both for-profit and non-profit private organizations, and at private clinics and hospitals.

Studies on HIV testing in Thailand reflect the concentrated nature of the epidemic in the country [7, 8]. They have mostly focused on the most at-risk populations (both youth and adults). These include men who have sex with men (MSM); female and male sex workers; and people who inject drugs (PWID) [9–13]. Very few studies have examined HIV testing at the overall population level [14–16]. Thus far, records of studies focusing specifically on the general Thai youth remain extremely scarce [17].

Young Thais are a particularly vulnerable population when it comes to HIV infection. There exists strong evidence showing that they engage in behavioral patterns that increase their risk of HIV infection. For example, the Bureau of Epidemiology [18] and the Ministry of Social Development and Human Security [19] have reported an increasing trend of unintended pregnancies and sexually transmitted infections (STIs) among Thai adolescents over the past 15 years. This occurrence points to an increasing rate of unprotected sex—probably as a result of the failure of safe sex messages to reach the general Thai youth population. A recent population-based study conducted in Nonthaburi province in Thailand [20], supporting previous similar findings [14, 21, 22], indicates a profound change in sexual norms among young Thais. This is characterized by a decline in the age of sexual initiation and a shift in the typical sexual partner—away from commercial sex workers to boyfriends or girlfriends in committed romantic relationships. In addition, there is, on average, a larger number of lifetime sexual partners, and a greater social acceptance of adolescent premarital sex.

Young people comprise a heterogeneous group of individuals whose sexual behaviors and vulnerability to HIV infection vary widely [23]. Previous research conducted in urban Chiang Mai, Thailand, found that out-of-school young people had a higher prevalence of risky sexual behaviors than those enrolled in general school and university (the sample for this group was recruited from Non-formal Education Centers (NFEC) and public spaces in Chiang Mai City). Out-of-school young people were also more likely to be sexually experienced. They also had a lower mean age of sexual debut, and a larger number of lifetime sexual partners, in comparison with their counterparts who attended general school and university [21, 24, 25]. Despite the documented profile of risky sexual behaviors among out-of-school Thai young people, nothing is known about the prevalence and correlates of HIV testing in this out-of-school population. Also, the general literature on HIV testing in general for Thai youth remains remarkably scarce.

The present study endeavors to fill this gap by documenting the prevalence and correlates of HIV testing among out-of-school young people enrolled in NFEC in urban Chiang Mai, Thailand. Our investigation focuses on the socio-demographic, behavioral, and psychosocial factors associated with HIV testing.

## Methods

### Ethics statement

The study was approved by the Office of Research Ethics, Human Experimentation Committee of the Research Institute for Health Sciences, Chiang Mai University (Certificate of Ethical Clearance No. 5/2015). Participants were first informed about the study’s objectives; their roles; and their rights to give or not to give any information during the interview. Additional topics discussed with participants were confidentiality of the personal data and manner in which findings would be presented. Participants provided verbal informed consent. Verbal informed consent was selected in preference to written informed consent, based on the vulnerable nature of our study population. Another reason for this was to prevent potential harm to the participants that could result from a breach of confidentiality. This process of informed consent was deemed appropriate by the Office of Research Ethics. For participants who were under 18 years old, written informed consent was obtained from their guardians—after providing the guardians with all the information regarding the study.

### Study design, participants, & setting

This was a cross-sectional study conducted between June and September 2015. Our study participants were defined as “young people aged 15–24, enrolled in NFEC in urban Chiang Mai, Thailand. Urban Chiang Mai—also referred to as Chiang Mai city, has rapidly expanded and developed as the epicenter of technology, industry, and education of Northern Thailand. Therefore, the area attracts an increasing number of young people from the countryside and neighboring provinces in search of education and work opportunities. Urban Chiang Mai is organized into 16 sub-districts, each comprising one NFEC. Non-formal Education in Thailand—run by the office of the Non-Formal Education Commission of the Thai Ministry of Education—offers the opportunity to youth and to adults who are out of school to get basic education. Also, these youth have the chance to continue their higher education via the certificate they are provided with upon completion of the program [26]. Young people enrolled in NFEC are provided with a three-hour tutorial class on a weekend basis. They may attend a class on Saturday and/or Sunday, and they may also attend a morning or afternoon program. The type of class they enroll in depends on their level of previous education. They will select a class at either primary, secondary, or high school level.

Our participants were recruited from all the 16 NFEC of Chiang Mai City. The procedure was that all age-eligible youths present on a teaching day were invited to participate—after having the survey explained to them. Our field research team included young investigators with extensive training both in field research and in quantitative and qualitative research methods.

### Survey Instrument

A structured, self-administered questionnaire was developed by the survey team. The questionnaire included 73 items, and was designed to address issues related to sexual and reproductive health of young people. It included items on participants’ socio-economic and demographic characteristics; recreational activities; alcohol, tobacco, and drug use; relationships; sexual identity and experience; sexually transmitted infections; birth control, pregnancy and abortion; need for sexual health services; and HIV testing.

While most questionnaire items were directly obtained from the literature [27, 28], other items—especially those related to HIV testing—were designed to fit the objectives of the current study. The questionnaire was first pre-tested. It was then refined in accordance with the test results—in order to ensure the clarity of the items. Participants completed the questionnaire in the classroom with desks spaced far enough to ensure privacy. Neither teachers nor any school-affiliated staff members were present while the students were completing the questionnaire. On average, it took 30 minutes to complete the questionnaire. The current study exclusively focuses on HIV testing.

### Variables

The outcome variable of interest was the past HIV testing status. This was assessed using the item “Have you ever been tested for HIV?” Firstly, the covariates included socio-demographic variables: age; sex; living status; employment status; and whether or not one currently has a boyfriend/girlfriend. The next category of variables was behavioral factors: “ever had sex”; “history of STI”, “ever been pregnant or made someone pregnant”, “number of lifetime sexual partners”, and “consistent use of condom”—specifically defined as using a condom for every act of sexual intercourse. The third category of variables was psychosocial variables. Among these, the first one was one’s self-efficacy of HIV testing—exemplified by the sentence, “I think I am able to get tested for HIV.” The second factor was one’s attitude toward HIV testing—to what degree did each participant think that “Getting tested for HIV is a responsible thing to do.”? Other psychosocial variables were subjective norms about HIV testing. Examples of positive norms included ideas such as “My family [parents, siblings] find it important that I have myself tested for HIV frequently.” Other examples were “My friend(s) find(s) it important that I have myself tested for HIV frequently.” Additional related variables were one’s perceived risk of HIV and one’s perceived risk of STIs and one’s degree of fear of HIV test results. The final variable was the perceived ease or difficulty of finding a nearby location to test for HIV.

### Statistical Analysis

The analysis was performed using SPSS (PASW) for Windows 17 (SPSS Inc., Chicago, Illinois, USA). Univariate analysis was used to obtain descriptive statistics of the selected variables. Chi-square was performed to document the associations of categorical covariates with the outcome of interest “ever tested for HIV” in the bivariate analysis. Multiple logistic regression was used to obtain adjusted odds ratios (AOR) and 95% confidence intervals (CI). Descriptive statistics were provided for the entire sample; however, bivariate and multivariate analyses were performed specifically in the subgroup of participants who were sexually active.

Two models were specified in the multiple logistic regression analysis. The first model included all the covariates. The second model included variables identified in the bivariate analysis with a P ≤ 0.10, and the variable “sex,” which was considered epidemiologically important. The diagnostic procedures yielded no evidence of multicollinearity.

## Results

### Participant characteristics

A total of 519 participants were recruited, and none of them declined to participate. First considering the demographic characteristics, the median age was 19 years [Interquartile range (IQR): 17.0–21.2]. The marital status of all participants was single. Slightly over half of them were female (53.2%); had work with income (56.3%); reported currently having a boyfriend or girlfriend (53.0%); and reported previously ever having had sex (56.8%).

Among those who were sexually active, 42% reported a history of STI—with self-reported symptoms or diagnosed by medical personnel. Among the total, 22% had ever been pregnant or made someone pregnant—of which 23.1% had ended up with an abortion or miscarriage. Also, 15.6% initiated sex before age 15. A substantial proportion had at least 2 lifetime partners (61.7%), and did not use condoms consistently (65.4%). The proportion of participants who reported ever having had an HIV test was 18.3% in the entire group. Among those who were sexually active, it was 27.8% (see Table 1).

**Table 1:** Socio-Demographic & Behavioral Characteristics of Participants.

	N = 519	%
**Sex**		
Male	243	46.8
Female	276	53.2
**Age**		
14–19 years	276	53.2
20–25 years	218	42.0
Missing	25	4.8
*Median (IQR)*	*19 (17–21*.*25)*	
**Living status**		
Living at home	223	43.0
Renting/Dormitory/other	293	56.5
Missing	3	0.6
**Employment with income**		
No	227	43.7
Yes	292	56.3
**Having boy/girlfriend**		
No	275	53.0
Yes	221	42.6
Missing	23	4.4
**Ever had sex**		
No	218	42.0
Yes	295	56.8
Missing	6	1.2
**Ever tested for HIV testing**		
No	390	75.1
Yes	95	18.3
Missing	34	6.6
**Ever tested for HIV**^#^		
No	200	67.8
Yes	82	27.8
Missing	13	4.4
**History of STI**^#^		
No	165	55.9
Yes	124	42.0
Missing	6	2.0
**Ever pregnant or made someone pregnant**^#^		
No	194	65.8
Yes	65	22.0
Missing	36	12.2
**Sexual debut**^#^		
< 15 years	46	15.6
≥ 15 years	239	81.0
Missing	10	3.4
**Number of life time sexual partners**^#^		
1 partner	86	29.2
≥ 2 partners	182	61.7
Missing	27	9.2
**Consistent condom use**^#^		
No	193	65.4
Yes	83	28.1
Missing	19	6.4

IQR, interquartile range;

^#^, data restricted to the subgroup of sexually active youth.

### Factors associated with ever tested for HIV

Table 2 shows the association of socio-demographic characteristics, behavioral factors, and psychosocial variables with ever tested for HIV. The bivariate analysis indicated that being female, aged 20–25 years, ever been pregnant or made someone pregnant, having two or more lifetime sexual partners, perception that testing for HIV is a responsible thing to do, perception that it is easy to find a location nearby to get tested for HIV, fear of HIV test result, and self-efficacy of HIV testing were significantly associated with increased odds of ever tested for HIV.

**Table 2:** Bivariate associations of socio-demographic, behavioral, and psychosocial factors with “ever tested for HIV” among sexually active participants.

	Ever Tested for HIV			Crude OR (95% CI)	*P* value^a^
	Yes	No	Total		
	n (%)	n (%)	n (%)		
**Sex**					
Male	34 (41.5)	110 (55.0)	144 (51.1)	1.00	
Female	48 (58.5)	90 (45.0)	138 (48.9)	1.72 (1.02–2.90)	0.039
**Age**					
14–19 years	24 (30.4)	93 (48.4)	117 (43.2)	1.00	
20–25 years	55 (69.6)	99 (51.6)	154 (56.8)	2.15 (1.23–3.75)	0.006
**Living status**					
Living at home	36 (44.4)	97 (48.5)	133 (47.3)	1.00	
Renting/Dormitory/other	45 (55.6)	103 (51.5)	148 (52.7)	1.71 (0.61–4.76)	0.537
**Employment with income**					
No	27 (32.9)	79 (39.5)	106 (37.6)	1.00	
Yes	55 (67.1)	121 (60.5)	176 (62.4)	1.33 (0.77–2.28)	0.301
**Currently having boy/girlfriend**					
No	18 (22.8)	53 (27.3)	71 (26.0)	1.00	
Yes	61 (77.2)	141 (72.7)	202 (74.0)	1.27 (0.69–2.35)	0.439
**History of STI**^**&**^					
No	45 (54.9)	112 (57.4)	157 (56.7)	1.00	
Yes	37 (45.1)	83 (42.6)	120 (43.3)	1.11 (0.66–1.86)	0.695
**Ever pregnant or made someone pregnant**					
No	38 (52.1)	151 (84.4)	189 (75.0)	1.00	
Yes	35 (47.9)	28 (15.6)	63 (25.0)	4.96 (2.69–9.15)	< 0.001
**Sexual debut**					
< 15 years	15 (19.2)	26 (13.3)	41 (15.0)	1.00	
≥ 15 years	63 (80.8)	169 (86.7)	232 (85.0)	0.64 (0.32–1.29)	0.218
**Number of life time sexual partners**					
1 partner	16 (21.9)	68 (36.6)	84 (32.4)	1.00	
≥ 2 partners	57 (78.1)	118 (63.4)	175 (67.6)	2.05 (1.09–3.85)	0.024
**Consistent condom use**					
No	60 (74.1)	124 (67.8)	184 (69.7)	1.00	
Yes	21 (25.9)	59 (32.2)	80 (30.3)	0.73 (0.41–1.32)	0.303
**Testing for HIV is a responsible thing to do**					
No	6 (7.3)	46 (23.8)	52 (18.9)	1.00	
Yes	76 (92.7)	147 (76.2)	223 (81.1)	3.96 (1.62–9.69)	0.001
**Finding a location nearby to get HIV test is…**					
Difficult	5 (6.1)	43 (21.7)	48 (17.1)	1.00	
Easy	58 (70.7)	98 (49.5)	156 (55.7)	5.09 (1.90–13.58)	0.001
Not sure	19 (23.2)	57 (28.8)	76 (27.1)	2.86 (0.99–8.28)	0.052
**I fear the result of HIV test**					
No	64 (78.0)	99 (49.7)	163 (58.0)	1.00	
Yes	14 (17.1)	60 (30.2)	74 (26.3)	0.36 (0.18–0.69)	0.003
Not sure	4 (4.9)	40 (20.1)	44 (15.7)	0.15 (0.05–0.45)	0.001
**I think I am able to get tested for HIV**					
No	5 (6.1)	43 (21.9)	48 (17.3)	1.00	
Yes	59 (72.0)	91 (46.4)	150 (54.0)	5.57 (2.08–14.89)	0.001
Not sure	18 (22.0)	62 (31.6)	80 (28.8)	2.49 (0.86–7.23)	0.092
**My family (parents, siblings) find it important I have myself tested for HIV frequently**					
No	25 (30.9)	69 (35.6)	94 (34.2)	1.00	
Yes	33 (40.7)	58 (29.9)	91 (33.1)	1.57 (0.84–2.93)	0.158
Not sure	23 (28.4)	67 (34.5)	90 (32.7)	0.94 (0.49–1.83)	0.872
**My friends find it important I have myself tested for HIV frequently**					
No	27 (33.3)	80 (41.2)	107 (38.9)	1.00	
Yes	34 (42.0)	55 (28.4)	89 (32.4)	1.83 (0.99–3.37)	0.052
Not sure	20 (24.7)	59 (30.4)	79 (28.7)	1.00 (0.51–1.96)	0.990
**HIV risk perception**					
No	57 (70.4)	140 (72.5)	197 (71.9)	1.00	
Yes	24 (29.6)	53 (27.5)	77 (28.1)	1.11 (0.62–1.97)	0.716
**STI risk perception**					
No	51 (63.0)	125 (64.8)	176 (64.2)	1.00	
Yes	30 (37.0)	68 (35.2)	98 (35.8)	1.08 (0.63–1.85)	0.776

OR, odds ratio; CI, confidence interval;

^a^ *P* values based on chi-square test of proportions unless otherwise specified; STI, sexually transmitted infection; &, referred to both diagnosed and self-reported symptoms of sexually transmitted infections.

We specified two models for the multiple logistic regressions. Overall, these models displayed similar results. Of the two models, Model 2 presented estimates with better precision; therefore, it was the one selected in the current report. (Table 3). The odds of “ever had HIV” was higher for participants who had “ever been pregnant or made someone pregnant;” who “had at least 2 lifetime sexual partners;” and who perceived that it is “easy to find a location nearby to test for HIV.” On the other hand, the odds were lower for those who “feared” or were not sure if they feared HIV test results. Regarding self-efficacy for HIV test, participants who perceived they were able to get tested for HIV and those who were uncertain, were more likely to ever had an HIV test than participants who did not have such a perception.

**Table 3:** Multivariate analysis of factors associated with ever tested for HIV among sexually active participants.

	Models Adjusted OR (95%CI)	
	(1)	(2)
**Sex** Female (vs Male)	2.50 (0.88–7.08)	2.03 (0.88–4.67)
**Age** 20–25 years (vs. 14–19 years)	1.20 (0.45–3.18)	1.03 (0.47–2.25)
**Employment with income** Yes (vs. none)	1.07 (0.41–2.78)	
**Living status** Living at home (vs. renting/dormitory/other)	1.70 (0.65–4.45)	
**Currently having boy/girlfriend** Yes (vs. no)	0.57 (0.20–1.62)	
**Testing for HIV is a responsible thing to do** Yes (vs no)	1.57 (0.39–6.36)	2.18 (0.65–7.31)
**Finding a location nearby to get an HIV test is…**		
Difficult	1.00	1.00
Easy	5.01 (1.11–22.68)*	4.67 (1.21–18.06)*
Not sure	4.07 (0.71–23.12)	3.31 (0.73–14.88)
**I fear the results of HIV testing**		
No	1.00	1.00
Yes	0.11 (0.03–0.38)^†^	0.21 (0.08–0.57)**
Not sure	0.09 (0.02–0.45)**	0.11 (0.02–0.47)**
**I think I am able to get tested for HIV**		
No	1.00	1.00
Yes	12.65 (2.10–76.26)**	4.92 (1.22–19.73)*
Not sure	17.08 (2.50–116.47)**	4.71 (1.01–21.92)*
**My family (parents, siblings) find it important that I have myself tested for HIV frequently**		
No	1.00	
Yes	1.32 (0.22–7.79)	
Not sure	0.57 (0.11–2.95)	
**My friends find it important that I have myself tested for HIV frequently**		
No	1.00	1.00
Yes	1.25 (0.25–6.32)	1.82 (0.75–4.41)
Not sure	0.97 (0.19–4.96)	1.01 (0.38–2.69)
**History of STI**^**&**^ yes (vs no)	0.56 (0.22–1.40)	
**HIV risk perception** yes (vs no)	2.84 (0.68–11.79)	
**STI risk perception** yes (vs no/don’t know)	0.52 (0.14–1.86)	
**Ever pregnant or made someone pregnant** yes (vs no)	6.34 (2.24–17.91)^†^	4.11 (1.76–9.60)**
**Sexual debut** ≥ 15 years (< 15 years)	0.48 (0.13–1.79)	
**Number of lifetime sexual partners** ≥ 2 partner (vs 1 partner)	2.59 (0.84–7.96)	2.63 (1.10–6.27)*
**Consistent condom use** yes (vs no)	1.17 (0.44–3.08)	

Adjusted OR, Adjusted odds ratio; CI, confidence interval;

* *P* value < 0.05;

** *P* value < 0.01;

^*†*^*P* value <0.001; STI, sexually transmitted infection; &, referred to both diagnosed and self-reported symptoms of sexually transmitted infections.

## Discussion

To our knowledge, this study is the first to examine HIV testing and its correlates in the population of out-of-school Thai youth attending the NFEC in Chiang Mai, Thailand. Our study revealed a significantly low prevalence of HIV testing—coupled with a high prevalence of risky sexual behaviors among our participants. Respectively, 65.4%, 61.7%, and 27.8% of sexually active young people did not consistently use condoms, had at least 2 lifetime partners; and reported ever having had been tested for HIV. The low HIV testing rate—coupled with the high prevalence of risky sexually behaviors—nurture a perfect environment where HIV can continue to be transmitted between partners who are in ignorance of their situation. It is also alarming to note that—despite the highly reported history of STIs (43.3%), which also reflect the low prevalence condom use—the majority of participants did not perceive themselves to be at risk for HIV infection. Additionally, what is of particular importance is the fact that no association was found between HIV testing and history of STIs or condom use. This finding signals a lack of concern about HIV infection among out-of-school Thai youth attending the NFEC.

As a result, there is a great need to rapidly develop evidence-based, youth-friendly strategies likely to improve HIV testing and to decrease risky sexual behaviors among out-of-school young Thai enrolled in NFEC in Chiang Mai, and Thailand at large. Such interventions should particularly be built on the correlates of HIV testing—such as those documented in this study. We found that the perceptions that it is easy to find a location nearby to test for HIV—and of self-efficacy for HIV testing—were associated with a high likelihood of “ever having had an HIV test.” Other factors associated with having had an HIV test were ever having been pregnant or made someone pregnant and having two or more lifetime sexual partners. On the other hand, fear of HIV test results was associated with decreased odds of ever having been tested for HIV.

The finding that participants who perceived it was easy to find a location nearby to test for HIV were more likely to report ever had an HIV test has two important implications. Firstly, it may tacitly imply that alleviation of distance as a structural barrier to accessing HIV testing sites could possibly improve HIV testing behavior among the young people. The second implication is that—in addition to addressing barriers such as distance—there is a need to ensure that young people are knowledgeable as to the availability of HIV testing services. This could take place, for example, through sensitization campaigns. This is particularly important because, in studies conducted in other settings, the lack of knowledge of service availability, and/or the lack of knowledge of the closest HIV testing site—rather than the actual unavailability of services—have been identified as barriers to HIV testing [29, 30].

We found that self-efficacy for HIV testing was associated with increased odds of “ever having been tested for HIV.” It is most likely that, in our study, self-efficacy for HIV testing is the outcome of a previous HIV testing experience, rather than being causal to it. This suggests that past experience of HIV testing—by enhancing self-efficacy—may be a facilitator for future HIV testing behavior. This is very important, considering that HIV testing should be regarded as a continuous behavior over the human life-course, rather than as a one-time event. In a previous study, self-efficacy was identified as strong predictor of willingness to test for HIV. However, in this study, self-efficacy was a complex concept based on people’s ability to engage in abstinence; remain faithful; and negotiate condom use. In our study, on the other hand, self-efficacy was considered to be a direct measure of people’s ability to go for an HIV test as in our study [31].

In line with other previously conducted studies [30, 32, 33], we have found that fear of HIV test results negatively impacts on the HIV testing behavior of young people. Interventions that address fear of HIV test results would be likely to improve HIV testing. However, the success of such interventions will largely depend on our understanding of drivers of fear of HIV testing. Many studies have linked fear of HIV test results to HIV risk perception in such a way that individuals who are perceived to be at risk for HIV as a result of their sexual behavior feared a positive HIV test result. Therefore, these people were more reluctant to go for an HIV test [34–36]. However, such a link was not established in our study. Both HIV and STI risk perception were neither associated with fear of HIV test result nor with ever had tested for HIV. Other studies have attributed fear of HIV test results—particularly, of positive test results—to a number of factors. These possible factors have included doubts about the availability and effectiveness of HIV medication; perceived stigma and discrimination accompanying a positive HIV status; and perceived lack of support from friends, family, and the community [32, 37]. In the case of Thailand, more studies are needed to unveil the context-specific factors underlying fear of HIV test results among the young in general—and specifically among out-of-school Thai youth attending the NFEC.

Participants who ever had been pregnant or made someone pregnant had increased odds of ever having had an HIV test in our study. Our findings support results from a previous study which found “ever been pregnant” as the highest predictor for HIV testing uptake among young people in South Africa [38]. A well-established explanation is that pregnant women—through the antenatal clinic—are more likely to undergo HIV testing under the Provider Initiated HIV Testing and Counseling (PIHTC) service delivered in the context of the prevention of mother-to-child transmission of HIV (PMTCT). Through the same context of PMTCT, male partners who made someone pregnant are also more likely to undergo HIV testing [38–41]. The increased contact of females with the health care system through antenatal clinics and other reproductive health services in many settings has been at the center of the generally observed gender differential in HIV testing, with females being more likely to test for HIV than males.

Although not significant, in our study, there was a notable trend for female participants to more likely report ever had an HIV test—compared to male participants. Our findings that the odds of reporting ever tested for HIV was higher among participants who had a larger number of lifetime partners—and among those who had ever had sex—were previously documented in other settings [42–45]. It is not obviously clear what factors mediate these associations in the context of our study. The mediating effect of HIV/STI risk perception on the association between sexual risk behaviors and HIV testing, documented elsewhere [32, 46–48], was not established in our study. This remains an open issue that future research will endeavor to clarify.

Our study also highlights the vulnerability to HIV infection and STIs of out-of-school Thai youth enrolled in NFEC—by showing both low levels of HIV testing and high risk sexual behaviors in this population. A prior research in the same population in urban Chiang Mai found that sexual risk behaviors were much more prevalent among out-of-school Thai youth—compared to their counterparts engaged in formal education [21, 24, 25]. Our study has identified correlates of HIV testing which can importantly inform future interventions aiming to improve HIV testing among Thai youth attending NFEC.

There is increasing support for a holistic approach to sexual and reproductive health for young people [49–51]. This approach should include interventions of varying nature (school-based, mass media, etc.), designed based on relevant contextual factors. These interventions should address a range of outcomes (HIV testing, sexual behaviors, HIV/AIDS knowledge) at different levels (individual, community, structural, etc.). This approach is recommended, as opposed to isolated interventions singling out one specific outcome. School-based interventions—which have been shown to be effective in increasing knowledge and decreasing HIV risk behaviors among youth [23]—potentially could enhance HIV testing among Thai youth enrolled in NFEC under the following circumstances.

First, they should be delivered in the form of health education and life skills programs. These programs should include counseling addressing factors such as fear of HIV testing results; self-efficacy; and HIV/STI risk perception. They should be coupled with interventions that promote access to HIV testing services both from a legal perspective (such as parental consent for HIV testing for adolescents aged under age 18) and a structural perspective (such as service availability and distance to testing sites).

A number of limitations to this study need to be acknowledged. Our study is cross-sectional by nature; thus, this design prohibits any causal inference. There was some risk of a “social desirability” bias in the data—given the sensitivity inherent to sexual health topics. However, the fact that we used young, well-trained investigators who could relate well to the study population might have minimized this bias. There was also a fair amount of missing values across all the covariates which could affect the results of our study.

One important limitation includes the fact that the variable “sexual identity” was not reliably collected so as to allow its inclusion in the analysis. “Sexual identity” in our study is a derived variable from two variables (gender of the participant & gender of the partner), and had important flaws. Firstly, the item on the gender of partner was restricted to the subsample of participants who stated that they currently had a partner. However, 26% of participants who were sexually active in general did not currently have a boyfriend or girlfriend. Secondly, the derived variable “sexual identity” does not explicitly tell how the participants identify themselves in terms of their sexual orientation. In addition, the lack of data on types of sexual intercourse (male-to-male; male-to-female; anal versus vaginal, etc.) limits the interpretation of risk in our study. This is particularly relevant because of the very high HIV risk documented among young MSM, male sex workers, and transgenders in Thailand [52, 53]. It is also important to note that the single items used to measure psychosocial variables -such as attitudes to HIV testing and self-efficacy—may not have captured well the various dimensions of those constructs. Future studies should use full and validated scales for our population of interest. The variable “History of STI” includes both actual STI diagnoses and self-reported symptoms of STIs. Self-reported symptoms are not accurate measures of STI—because genital infections may also be caused by non-sexually transmitted conditions. Lastly, although the results of this study—to a large extent—represent the situation of young people enrolled in NFE in Chiang Mai, it is not clear to what extent they can be generalized to young people enrolled in NFE in other provinces of Thailand and/or to out-of-school young people who do not attend NFE.

In summary, we found that a substantially high proportion of Thai youth who engaged in risky sexual behavior, yet reported low rates of ever having been tested for HIV. We were able to identify a number of individual-level factors (such as fear of HIV test results and perception that it is easy to locate an HIV testing site nearby) which can serve as useful guidelines for future interventions to enhance HIV testing uptake among young people enrolled in NFEC in Thailand. Such interventions should, however, consider the broader contextual and structural landscape within which young people live.

## Supporting Information

S1 Dataset Dataset of the study. (SAV)

S1 Questionnaire Questionnaire Thai version. (DOC)

S2 Questionnaire Questionnaire English Version. (DOCX)
