# Supplementary material for: Prevalence and Correlates of HIV Testing among Young People Enrolled in Non-Formal Education Centers in Urban Chiang Mai, Thailand: A Cross-Sectional Study
Source: PLoS One. 2016 Apr 12;11(4):e0153452. doi: 10.1371/journal.pone.0153452 (PMC4829184; doi:10.1371/journal.pone.0153452)
Supplement: S1 Questionnaire — (DOC) [file pone.0153452.s002.doc]

เลขที่แบบสอบถาม    

[สำหรับผู้วิจัย]

| **แบบสอบถามวิถีชีวิตวัยรุ่นที่ศึกษาในศูนย์การศึกษานอกระบบ  และการศึกษาตามอัธยาศัยในเมืองเชียงใหม่**  **วิธีการตอบคำถาม:**  ให้นักเรียนทำเครื่องหมายถูก 🗸 ลงในช่อง 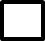 หรือเติมตัวเลข หรือข้อความ ลงในช่องว่าง (________) หากไม่ต้องการให้คำตอบ ให้ทำเครื่องหมายถูก 🗸 ลงในช่อง 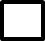 ไม่ตอบ |
| --- |

**ตอนที่ 1: ข้อมูลทางด้านสังคมประชากร**

วันที่ตอบแบบสอบถาม (วันที่-เดือน-พ.ศ.)   -   -  

1.1 ท่านเป็นเพศใด?


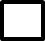
 1.ชาย
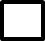
 2.หญิง

1.2 ท่านมีอายุเท่าไหร่ เมื่อวันครบรอบวันเกิดที่ผ่านมา?   ปี

1.3 ท่านนับถือศาสนาอะไร?


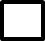
 1.พุทธ
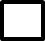
 2.คริสต์
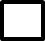
 3.อิสลาม


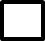
 4.ไม่นับถือศาสนาใด
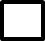
 5.อื่น ๆ (โปรดระบุ) ___________________

1.4 ตั้งแต่ท่านเกิดจนถึงอายุ 15 ปี ท่านใช้ชีวิตส่วนใหญ่ที่ไหน?


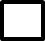
 1.ในประเทศไทย
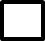
 2.นอกประเทศไทย (โปรดระบุ) _______________________

1. 5 ท่านเป็นชาติพันธุ์กลุ่มใด? **(ตอบได้มากกว่า 1 ข้อ)**


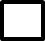
 1.จีน
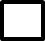
 2.พม่า
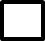
 3.ไทใหญ่


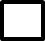
 4.ไทยภูเขา (ระบุ)____________
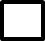
 5.ไทยพื้นราบ
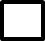
 6.อื่นๆ (ระบุ)____________

1.6 ปัจจุบันท่านพักอาศัยอยู่ที่ใด?


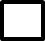
 1.อาศัยอยู่บ้านของตนเอง
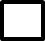
 2.อาศัยอยู่บ้านเช่า/หอพักนอกโรงเรียน


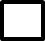
 3.อาศัยอยู่หอพักในโรงเรียน
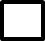
 4.อื่น ๆ (ระบุ) _____________________

1.6.1 ปัจจุบันท่านพักอาศัยอยู่กับใคร? **(ตอบได้มากกว่า 1 ข้อ)**


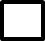
 1.อาศัยอยู่กับพ่อแม่/ ญาติ
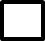
 2.อาศัยอยู่กับนายจ้าง


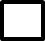
 3.อาศัยอยู่กับเพื่อน
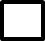
 4.อาศัยอยู่คนเดียว


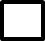
 5.คู่สมรส/คู่รัก (แฟน)
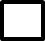
 6.อื่น ๆ (โปรดระบุ) __________

1.7 ขณะนี้ท่านยังเรียนหนังสืออยู่หรือไม่ ?


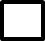
 1.เรียนในสถานศึกษาของรัฐหรือเอกชน
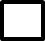
 2.เรียน กศน.


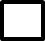
 3.ไม่เรียน (**ข้ามไปข้อ 1.8**)
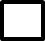
 4.อื่นๆ (ระบุ)________________

1.7.1 ปัจจุบันท่านกำลังศึกษาอยู่ระดับชั้นใด?


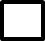
 1. ประถมศึกษา
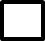
 2. มัธยมศึกษาตอนต้น
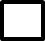
 3. มัธยมศึกษาตอนปลาย

1.8 ขณะนี้ท่านมีงานทำที่ได้รับเงินค่าจ้างหรือไม่?


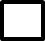
 1.ไม่มี
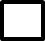
 2.มี (ระบุ) ________________ บาท/เดือน

**ตอนที่ 2: ข้อมูลเกี่ยวกับวิถีชีวิต**

2.1 ขณะนี้ท่านหรือคนในครอบครัวมีสิ่งของต่อไปนี้หรือไม่? (**ตอบได้มากกว่า 1 ช่อง**)

|  | **ฉันมีเป็นของตนเอง** | **ครอบครัวของฉันมี** | **ฉันและครอบครัวไม่มีสิ่งนี้** |
| --- | --- | --- | --- |
| โทรศัพท์มือถือ | 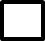 | 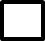 | 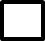 |
| อินเตอร์เน็ต (ในมือถือ/เครื่องคอมพิวเตอร์) | 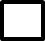 | 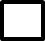 | 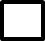 |
| โทรทัศน์ | 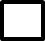 | 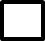 | 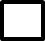 |
| รถจักรยานยนต์ | 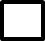 | 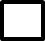 | 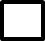 |
| รถปิ๊กอัพ/รถเก๋ง | 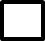 | 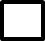 | 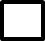 |

2.2 ในระยะ **1 ปี ที่ผ่านมา** ท่านได้ดื่มเครื่องดื่มที่มีแอลกอฮอล์ หรือไม่?


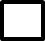
 1.ดื่ม
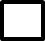
 2.ไม่ดื่ม **ถ้าไม่ดื่ม ให้ข้ามไปข้อ 2.3**

2.2.1 ถ้าดื่ม ท่านดื่มเครื่องดื่มที่มีแอลกอฮอล์บ่อยแค่ไหน?


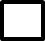
 1.ดื่มนาน ๆ ครั้ง
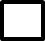
 2.ดื่มสัปดาห์ละครั้ง
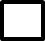
 3.ดื่มมากกว่าสัปดาห์ละครั้ง

2.2.2 โดยเฉลี่ยแล้วท่านดื่มเครื่องดื่มที่มีแอลกอฮอล์ครั้งละกี่แก้ว/ก๊ง?

- 1. 1-2 แก้ว/ก๊ง
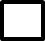
 2. 3-5 แก้ว/ก๊ง
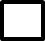
 3. มากกว่า 5 แก้ว/ก๊ง

2.3 ในระยะ **1 ปี ที่ผ่านมา** ท่านได้สูบบุหรี่หรือไม่?


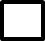
 1.สูบ
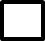
 2.ไม่สูบ **ถ้าไม่สูบ ข้ามไปข้อ 2.4**

2.3.1 โดยเฉลี่ยแล้วท่านสูบบุหรี่บ่อยแค่ไหน?


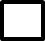
 1. สูบนาน ๆ ครั้ง
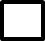
 2. 1-5 มวนต่อวัน


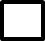
 3. 6-10 มวนต่อวัน
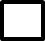
 4. มากกว่า 10 มวนต่อวัน

2.4 ในระยะ **1 ปี ที่ผ่านมา** ท่านได้เสพสิ่งต่อไปนี้หรือไม่ อย่างไร?

|  | **เสพประมาณ** | **เสพนาน ๆ ครั้ง** | **ไม่ได้เสพสิ่งนี้** |
| --- | --- | --- | --- |
| ยาบ้า | __ __ ครั้ง/เดือน | 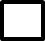 | 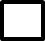 |
| กัญชา | __ __ ครั้ง/เดือน | 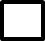 | 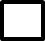 |
| สารระเหย | __ __ ครั้ง/เดือน | 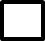 | 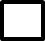 |
| ยาไอซ์ | __ __ ครั้ง/เดือน | 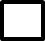 | 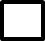 |
| เฮโรอีน (สูบ) | __ __ ครั้ง/เดือน | 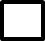 | 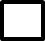 |
| ฉีดสารเสพติดเข้าเส้นเลือด | __ __ ครั้ง/เดือน | 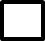 | 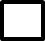 |
| อื่น ๆ (ระบุ) _____________ | __ __ ครั้ง/เดือน | 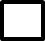 | 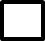 |

**ตอนที่ 3: ข้อมูลเกี่ยวกับความรักความสัมพันธ์**

**0**

3.1 ในระยะ **1 ปี ที่ผ่านมา** ท่านใช้เวลาว่างส่วนใหญ่อยู่กับใคร? **(ตอบได้มากกว่า 1 ข้อ)**


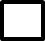
 1.เพื่อน
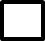
 2.แฟน
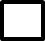
 3.มารดา


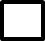
 4.บิดา
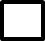
 5.พี่น้อง
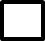
 6.ญาติ


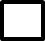
 7.อยู่กับตัวเอง

3.2 **ขณะนี้**ท่านมีแฟนหรือไม่?


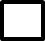
 1.มี
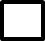
 2.ไม่มี **ถ้าไม่มี ให้ข้ามไปตอนที่ 4**

3.2.1 แฟนของท่านอายุเท่าไหร่? __ __ ปี

3.2.2 แฟนของท่านเป็นเพศชายหรือเพศหญิง?


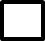
 1.ผู้ชาย
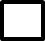
 2.ผู้หญิง

3.2.3 ท่านวางแผนจะแต่งงาน / มีความสัมพันธ์แบบถาวร กับแฟนคนนี้หรือไม่?


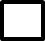
 1.ใช่
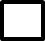
 2.ไม่ใช่
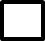
 3.ยังไม่แน่ใจ

3.2.4 ท่านเคยมีเพศสัมพันธ์กับแฟนคนนี้หรือไม่?


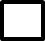
 1.เคย
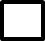
 2.ไม่เคย

3.2.5 ปัจจุบันท่านพักอาศัยอยู่กับแฟนใช่หรือไม่?


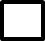
 1.ใช่
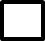
 2.ไม่ใช่

**ตอนที่ 4: ข้อมูลเกี่ยวกับเพศสัมพันธ์**

4.1 ท่านเคยมีเพศสัมพันธ์หรือไม่?

*[****เพศสัมพันธ์*** *หมายถึง อวัยวะเพศของคนใดคนหนึ่งสอดใส่เข้าไปในช่องคลอดหรือทวารหนักของอีกคนหนึ่ง]*


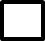
 1.เคย


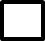
 2.ไม่เคย

**ถ้าไม่เคยมีเพศสัมพันธ์ ให้ข้ามไปตอบในตอนที่ 7**

4.2 ในชีวิตที่ผ่านมาท่านเคยมีเพศสัมพันธ์กับคนทั้งหมดกี่คน   คน

4.3 ท่านมีเพศสัมพันธ์**ครั้งแรก** เมื่ออายุเท่าไหร่? อายุ   ปี

- - 1. เพศสัมพันธ์**ครั้งแรก**เกิดขึ้นที่ไหน?


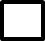
 1.ที่พักของฉัน
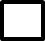
 2.ที่พักของคู่นอน

3.ที่พักของเพื่อน 4.โรงแรมหรือโมเต็ล

5.ในรถยนต์ 6.ในที่โล่งแจ้ง

7.ในสถานบริการทางเพศ 8.อื่น ๆ (โปรดระบุ) _______________

4.3.2 คู่นอน**คนแรก**ของท่านเป็นใคร?

1.แฟน 2.เพื่อน

3.ญาติ 4.คนรู้จัก

5.คนแปลกหน้า 6.ผู้ขายบริการทางเพศ

7.อื่น ๆ (โปรดระบุ) _______________

4.3.3 คู่นอน**คนแรก**ของท่านเป็นเพศชายหรือเพศหญิง?

1.ผู้ชาย 2.ผู้หญิง

4.3.4 ในเพศสัมพันธ์**ครั้งแรก** ท่าน/คู่นอนได้ใช้วิธีการป้องกันโรค / การตั้งครรภ์หรือไม่ อย่างไร? **(ตอบได้มากกว่า 1 ข้อ)**

1.หลั่งภายนอก 2.ถุงยางอนามัย

3.ใช้ยาคุมกำเนิดฉุกเฉิน 4.ใช้ยาพื้นบ้าน/ยาสมุนไพร

5.ไม่ได้ใช้วิธีใด ๆ 6.อื่น ๆ (โปรดระบุ) _______________

4.4 ท่านมีเพศสัมพันธ์**ครั้งสุดท้าย**เมื่อไหร่**?**

1. 1-2 วัน ที่ผ่านมา 2. ภายใน 1 สัปดาห์ ที่ผ่านมา

3. ภายใน 1 เดือน ที่ผ่านมา 4. ภายใน 3 เดือน ที่ผ่านมา

5. ภายใน 1 ปี ที่ผ่านมา 6. มากกว่า 1 ปี ที่ผ่านมา

4.4.1 ในเพศสัมพันธ์**ครั้งสุดท้าย**นั้น คู่นอนของท่านเป็นใคร?

1.แฟน 2.เพื่อน 3.ญาติ

4.คนรู้จัก 5.คนแปลกหน้า 6.ผู้ขายบริการทางเพศ

7.อื่น ๆ (โปรดระบุ) _______________

4.4.2 คู่นอน**ครั้งสุดท้าย**ของท่านเป็นชายหรือหญิง?

1.ผู้ชาย 2.ผู้หญิง

4.4.3 ในเพศสัมพันธ์**ครั้งสุดท้าย** ท่าน/คู่นอนได้ใช้วิธีการป้องกันโรค / การตั้งครรภ์หรือไม่ อย่างไร? **(ตอบได้มากกว่า 1 ข้อ)**

1.หลั่งภายนอก 2.ถุงยางอนามัย

3.ยาเม็ดคุมกำเนิดชนิดแผง 4.ใช้ยาคุมกำเนิดฉุกเฉิน

5.ใช้ยาพื้นบ้าน/ยาสมุนไพร 6.ไม่ได้ใช้วิธีใด ๆ

7.อื่น ๆ (โปรดระบุ) _______________

4.5 ท่าน**เคยถูกบังคับ** (ทางร่างกายหรือจิตใจ) ให้มีเพศสัมพันธ์โดยไม่ได้รับความยินยอมหรือไม่**?**

1.เคย 2.ไม่เคย **ถ้าไม่เคยให้ข้ามไปตอนที่ 5**

4.5.1 ท่านอายุเท่าไหร่เมื่อเหตุการณ์นั้นเกิดขึ้น**ครั้งแรก**? อายุ   ปี

4.5.2 เหตุการณ์นั้นของท่านเกิดขึ้นกับใคร?

1.แฟน 2.เพื่อน

3.ญาติ 4.คนรู้จัก

5.คนแปลกหน้า 6.อื่น ๆ (ระบุ) _______________

**ตอนที่ 5: *โรคติดต่อทางเพศสัมพันธ์***

5.1 ท่านเคยมีอาการ หรือเคยเป็นโรคต่าง ๆ ต่อไปนี้หรือไม่?

|  | **เคย** | **ไม่เคย** | **ไม่แน่ใจ / ไม่ทราบ** |
| --- | --- | --- | --- |
| มีแผลหรือตุ่มที่อวัยวะสืบพันธุ์ |  |  |  |
| ปวด/แสบ/ขัด เวลาปัสสาวะ |  |  |  |
| คันบริเวณอวัยวะสืบพันธุ์ |  |  |  |
| มีสารคัดหลั่งที่ผิดปกติ (เช่นมีหนอง หรือมีมูกสีเหลืองหรือเขียว) ออกจากอวัยวะสืบพันธุ์ |  |  |  |
| ช่องคลอด/ปากมดลูกอักเสบ (**เฉพาะเพศหญิง**) |  |  |  |
| ปีกมดลูกอักเสบ (**เฉพาะเพศหญิง**) |  |  |  |
| ท่อปัสสาวะอักเสบ |  |  |  |
| หนองใน |  |  |  |
| ซิฟิลิส |  |  |  |
| หนองในเทียม (คลาไมเดีย) |  |  |  |
| เริม |  |  |  |
| ติดเชื้อ เอช ไอ วี |  |  |  |
| อื่น ๆ (โปรดระบุ)___________ ___________ |  |  |  |

5.1.1 ถ้าเคย **ครั้งสุดท้าย**ที่ผ่านมา ท่านทำอย่างไร?

1.ไปสถานีอนามัยหรือโรงพยาบาลรัฐบาล 2.ไปคลินิกหรือโรงพยาบาลเอกชน

3.ไปร้านขายยา 4.หายาจากที่อื่นมารับประทานเอง

5.ไม่ทำอย่างไร 6.อื่น ๆ (โปรดระบุ) ______________

**ตอนที่ 6: การคุมกำเนิด การตั้งครรภ์ และการทำแท้ง**

6.1 ตั้งแต่ท่านมีเพศสัมพันธ์ครั้งแรกจนถึงปัจจุบัน ท่านและคู่นอนใช้วิธีการคุมกำเนิดต่อไปนี้หรือไม่อย่างไร?

|  | **ใช้ตลอด**  **เวลา** | **ใช้เป็นส่วนใหญ่** | **ใช้เป็นบางโอกาส** | **ไม่เคยใช้เลย** |
| --- | --- | --- | --- | --- |
| หลั่งภายนอก |  |  |  |  |
| นับระยะปลอดภัย |  |  |  |  |
| ใช้ถุงยางอนามัย |  |  |  |  |
| ยาเม็ดคุมกำเนิด |  |  |  |  |
| ยาคุมกำเนิดฉุกเฉิน |  |  |  |  |
| ยาฉีดคุมกำเนิด |  |  |  |  |
| ห่วงอนามัย |  |  |  |  |
| ยาฝังคุมกำเนิด |  |  |  |  |
| ยาพื้นบ้านหรือยาสมุนไพร |  |  |  |  |
| อื่น ๆ โปรดระบุ_________ |  |  |  |  |

6.5 (**กรณีผู้หญิง**) ท่านเคยตั้งครรภ์หรือไม่? (**กรณีผู้ชาย**) ท่านเคยทำให้คู่นอนของท่านตั้งครรภ์หรือไม่?

1.เคย 2.ไม่เคย **ถ้าไม่เคย ให้ข้ามไปตอนที่ 8**

6.5.1 ถ้าเคย เคยกี่ครั้ง?  ครั้ง

6.5.2 ผลของการตั้งครรภ์เป็นอย่างไรบ้าง? **(ตอบได้มากกว่า 1 ข้อ)**

1.ทำแท้ง 2.แท้งเอง 3.คลอด

4.กำลังตั้งครรภ์อยู่ 5.อื่น ๆ (โปรดระบุ) _________________

**ถ้าไม่เคยทำแท้ง ให้ข้ามไปตอนที่ 8**

6.5.3 ถ้าเคยทำแท้ง ท่านหรือคู่นอนของท่านเคยทำแท้งกี่ครั้ง?  ครั้ง

6.5.4 ท่านหรือคู่นอนของท่านทำอย่างไรบ้างตอน**ทำแท้งครั้งสุดท้าย** **(ตอบได้มากกว่า 1 ข้อ)**

1.ไปคลินิก/โรงพยาบาลเอกชน 2.ไปสถานบริการของรัฐ

3.ไปหาหมอเถื่อน 4.ให้เพื่อน/แฟนซื้อยาขับให้

5.จัดการเอง 6.ไม่ทราบ/ไม่แน่ใจ

7.อื่น ๆ (โปรดระบุ) _______________

6.5.5 ท่านหรือคู่นอนของท่านต้องใช้จ่ายเงินจำนวนเท่าไหร่ในการ**ทำแท้งครั้งสุดท้าย**?

1. ต่ำกว่า 1,000 บาท 2. 1,000 – 2,499 บาท

3. 2,500 – 4,999 บาท 4. 5,000 บาท ขึ้นไป

6.5.6 ผลของการ**ทำแท้งครั้งสุดท้าย** เป็นอย่างไร?

1.ไม่มีภาวะแทรกซ้อนใด

2.มีภาวะแทรกซ้อนเล็กน้อยและไม่ได้จัดการอะไร

3.มีภาวะแทรกซ้อนเล็กน้อยและได้ซื้อยาที่ร้านขายยา

4.มีภาวะแทรกซ้อนมากและได้ไปพบแพทย์

5.ไม่ทราบ/ไม่แน่ใจ

6.อื่น ๆ (โปรดระบุ) _______________

**เมื่อท่านตอบถึงตรงนี้แล้ว กรุณาข้ามไปตอบในตอนที่ 8**

**ตอนที่ 7: สำหรับผู้ที่ไม่มีประสบการณ์ทางเพศเท่านั้น**

***ผู้ที่มีประสบการณ์ทางเพศแล้ว กรุณาข้ามไปตอบในตอนที่ 8***

**ผู้คนอาจมีเหตุผลที่หลากหลายในการไม่มีเพศสัมพันธ์ คำถามต่อไปนี้จะถามท่านเกี่ยวกับความคิดเห็นของตัวท่านเกี่ยวกับเรื่องนี้**

7.1 เหตุผลที่ท่านไม่มีเพศสัมพันธ์

|  | **ใช่** | **ไม่ใช่** | **ไม่แน่ใจ** |
| --- | --- | --- | --- |
| ฉันรู้สึกยังไม่พร้อมที่จะมีเพศสัมพันธ์ |  |  |  |
| ฉันยังหาโอกาสไม่ได้ |  |  |  |
| ฉันคิดว่าการมีเพศสัมพันธ์ก่อนแต่งงานเป็นสิ่งที่ไม่ถูกต้อง |  |  |  |
| ฉันกลัวท้อง / กลัวทำคนอื่นท้อง |  |  |  |
| ฉันกลัวติดเอดส์ / กลัวโรคติดต่อทางเพศสัมพันธ์ |  |  |  |
| ฉันแคร์ความรู้สึกของพ่อแม่ |  |  |  |
| ฉันกลัวคนที่สถานศึกษา / ที่ทำงาน จะตำหนิหรือนินทา |  |  |  |
| อื่น ๆ (โปรดระบุ)____________________­­­­­­___ |  |  |  |

7.2 ในอนาคต ท่านคิดว่าจะมีเพศสัมพันธ์เมื่อใด? **(ตอบเพียงข้อเดียวที่ตรงใจมากที่สุด)**

1.ฉันจะรอจนถึงตอนแต่งงาน 2.ฉันจะรอจนถึงมีการหมั้นหมายกันก่อน

3.ฉันจะรอจนถึงเมื่อพบคนที่ฉันรัก 4.ฉันจะรอจนถึงเมื่อมีโอกาส

5.ฉันวางแผนที่จะไม่มีเพศสัมพันธ์กับใครเลย 6.ยังไม่แน่ใจในอนาคต

7.อื่น ๆ (โปรดระบุ) ________________________________

7.3 ท่านรู้สึก**ถูกกดดัน**ที่จะต้องมีเพศสัมพันธ์หรือไม่

(***ถูกกดดัน*** *หมายถึง* *การมีสิ่งอื่นจากภายนอกที่เข้ามามีอิทธิพลต่อการตัดสินใจมี/ อยากมีเพศสัมพันธ์)*

1.ใช่ ฉันถูกกดดันมาก 2.ใช่ แต่ฉันถูกกดดันเล็กน้อยเท่านั้น

3.ฉันไม่ถูกกดดันเลย **ถ้าไม่ถูกกดดันเลย ให้ข้ามไปตอนที่ 8**

7.3.1 ถ้าใช่ ท่านถูกกดดันจากใคร **(ตอบได้มากกว่า 1 ข้อ)**

1.แฟน 2.เพื่อนเพศเดียวกัน 3.เพื่อนต่างเพศ

4.สื่อต่าง ๆ 5.อื่น ๆ (โปรดระบุ) _________________

**ตอนที่ 8: การใช้บริการด้านสุขภาพทางเพศและอนามัยเจริญพันธุ์**

8.1 ท่านเคยไปสถานบริการสุขภาพ เพื่อรับบริการเกี่ยวกับโรคติดต่อทางเพศสัมพันธ์ การคุมกำเนิด การตั้งครรภ์ หรือการทำแท้ง หรือไม่?

1.เคย 2.ไม่เคย  **ถ้าไม่เคย ข้ามไปตอบข้อ 8.7**

8.2 ถ้าเคย ท่านเคยไปใช้บริการกี่ครั้ง? __ __ ครั้ง

8.3 ครั้งสุดท้ายที่ไปสถานบริการเกี่ยวกับสุขภาพด้านเพศและอนามัยเจริญพันธุ์นั้น ท่านไปใช้บริการเกี่ยวกับอะไร? **(ตอบได้มากกว่า 1 ข้อ)**

1.โรคติดต่อทางเพศสัมพันธ์ 2.การคุมกำเนิด

3.ตรวจการตั้งครรภ์ 4.ยุติการตั้งครรภ์

5.ฝากครรภ์/คลอด 6.อื่น ๆ (โปรดระบุ) _________________

8.4 ครั้งสุดท้ายที่ท่านไปใช้บริการ ท่านได้ใช้สถานบริการแห่งใด?

1.คลินิก/โรงพยาบาลเอกชน 2.สถานบริการของรัฐ

3.สถานบริการขององค์กรเอ็นจีโอ 4.อื่น ๆ (โปรดระบุ) _________________

8.5 เมื่อท่านไปใช้บริการในครั้งนั้น ท่านรู้สึกพึงพอใจกับการให้บริการของเจ้าหน้าที่ หรือไม่?

1.พึงพอใจ

2.ไม่ค่อยพึงพอใจ

8.6 ท่านคิดว่าวิธีการรักษาความลับของสถานบริการที่ท่านไปใช้บริการนั้นดีเพียงพอหรือไม่?

1.ดีเพียงพอ 2.ยังไม่ดีพอ 3.ไม่แน่ใจ

8.7 ท่านคิดว่าท่านต้องการให้มีคลินิก / สถานบริการสุขภาพทางเพศและอนามัยเจริญพันธุ์ เฉพาะสำหรับวัยรุ่น หรือไม่?

1.ต้องการ

2.ไม่ต้องการ

3.ยังไม่แน่ใจ / ต้องการข้อมูลเพิ่มเติม

**ตอนที่ 9 : เกี่ยวกับเรื่อง HIV/เอดส์**

**คำถามตอนสุดท้ายนี้จะถามท่านเกี่ยวกับการตรวจหาการติดเชื้อเอชไอวี/ เอดส์**

9.1 ท่านเคยได้รับการตรวจหาการติดเชื้อเอชไอวี/ เอดส์ หรือไม่ ?

1.เคย 2.ไม่เคย **ถ้าไม่เคย ข้ามไปตอบข้อ 9.3**

9.2 ท่านเคยได้รับการตรวจหาการติดเชื้อเอชไอวี/ เอดส์ในระยะ 12 เดือนที่ผ่านมาใช่หรือไม่ ?

1.ใช่ 2.ไม่ใช่

9.3 มีความเป็นไปได้มากที่ท่านจะเข้ารับตรวจหาการติดเชื้อเอชไอวี/ เอดส์ในโอกาสต่อไปหรือไม่ ?

1.เป็นไปได้น้อยมาก 2.เป็นไปได้น้อย 3.ค่อนข้างเป็นไปได้

4.เป็นไปได้มาก 5.ไม่แน่ใจ

9.4 การเข้ารับตรวจหาการติดเชื้อเอชไอวี/ เอดส์เป็นสิ่งที่แสดงถึงความรับผิดชอบที่ควรทำใช่หรือไม่ ?

1.ใช่ 2.ไม่ใช่

9.5 ท่านคิดว่าเป็นเรื่องยากลำบากในการหาสถานที่ตรวจหาการติดเชื้อเอชไอวี/ เอดส์หรือไม่ ?

1.ยากมาก 2.ยาก 3.ง่าย

4.ง่ายมาก 5.ไม่แน่ใจ

9.6 ท่านกลัวผลของการตรวจหาการติดเชื้อเอชไอวี/ เอดส์หรือไม่ ?

1.กลัว 2. ไม่กลัว 3.ไม่แน่ใจ

9.7 ท่านคิดว่าท่านสามารถไปตรวจหาการติดเชื้อเอชไอวี/ เอดส์ใช่หรือไม่ ?

1.ใช่ 2. ไม่ใช่ 3.ไม่แน่ใจ

9.8 ท่านคิดว่าท่านสามารถไปตรวจหาการติดเชื้อเอชไอวี/ เอดส์ ถึงแม้ท่านจะกลัวผลของการตรวจใช่หรือไม่ ?

1.ใช่ 2. ไม่ใช่ 3.ไม่แน่ใจ

9.9 **ครอบครัว**ของท่าน (พ่อแม่, พี่น้อง) สนับสนุนให้ท่านเข้ารับตรวจหาการติดเชื้อเอชไอวี/ เอดส์บ่อยๆ ใช่หรือไม่ ?

1.ใช่ 2. ไม่ใช่ 3.ไม่แน่ใจ

9.10 **คู่นอน**ของท่าน สนับสนุนให้ท่านเข้ารับตรวจหาการติดเชื้อเอชไอวี/ เอดส์บ่อยๆ ใช่หรือไม่ ?

1.ใช่ 2. ไม่ใช่ 3.ไม่แน่ใจ

9.11  **เพื่อน**ของท่าน สนับสนุนให้ท่านเข้ารับตรวจหาการติดเชื้อเอชไอวี/ เอดส์บ่อยๆ ใช่หรือไม่ ?

1.ใช่ 2. ไม่ใช่ 3.ไม่แน่ใจ

9.12 ท่านคิดว่า**ในขณะนี้**ท่านมีความเสี่ยงต่อการติดเชื้อเอชไอวี/ เอดส์หรือไม่ ?

1.เสี่ยงมาก 2.เสี่ยงปานกลาง 3.เสี่ยงน้อย 4.ไม่เสี่ยง

5.ไม่แน่ใจ

9.13 ท่านคิดว่า**ในขณะนี้**ท่านมีความเสี่ยงต่อการติดโรคติดต่อทางเพศสัมพันธ์หรือไม่ ?

1.เสี่ยงมาก 2.เสี่ยงปานกลาง 3.เสี่ยงน้อย 4.ไม่เสี่ยง

5.ไม่แน่ใจ

9.14 ท่านคิดว่าอะไรเป็นปัญหาอุปสรรค (ข้อยุ่งยาก) ของคนวัยรุ่น (วัยหนุ่มสาว) ในการเข้ารับการตรวจหาการติดเชื้อเอชไอวี/ เอดส์?

………………………………………………………………………………………………………………………………………………………………………………………………………………………………………………………………………………………………………………………………………………………

9.15 ท่านคิดว่าวิธีการใดต่อไปนี้ ที่จะทำให้ท่านตัดสินใจรับการตรวจหาการติดเชื้อเอชไอวี/ เอดส์มากที่สุด

1.ตรวจเองที่บ้าน โดยครอบครัวรับรู้ 2. สมัครใจไปตรวจที่ศูนย์บริการสุขภาพ

3.ตรวจเองที่บ้าน โดยไม่ให้คนอื่นรับรู้ 4. สมัครใจไปตรวจที่ศูนย์บริการเคลื่อนที่ในชุมชน

**ขอขอบคุณท่านเป็นอย่างยิ่ง ในการให้ความร่วมมือตอบแบบสอบถาม**
