# Supplementary material for: Prevalence and Correlates of HIV Testing among Young People Enrolled in Non-Formal Education Centers in Urban Chiang Mai, Thailand: A Cross-Sectional Study
Source: PLoS One. 2016 Apr 12;11(4):e0153452. doi: 10.1371/journal.pone.0153452 (PMC4829184; doi:10.1371/journal.pone.0153452)
Supplement: S2 Questionnaire — (DOCX) [file pone.0153452.s003.docx]

Study ID number _ _ _ _

*[For official use only]*

| **Questionnaire survey**  **Prevention of AIDS and Pregnancy among Young People Enrolling Non-formal Education Centers in Urban Chiang Mai, Thailand**  **How to answer:**   - Just put a tick in the space __ next to the appropriate answer like this **√**. - Or write in a number on a line __ __ like this **1 8**. - Not all the questions will apply to you, so follow instructions. - Please ask for help or explanations if you are not sure.   **Important:**   - It is very important to the study that you answer these questions completely honestly and as accurately as you can. - Some things may be hard to remember, so please take your time.   **Confidentiality:**   - The questions in this self-completion questionnaire are mostly very personal. - Your answers will be treated in strict confidence; the research team does not need to see them. - When you have finished, leave the questionnaire in a box provided. Your name will not be on the questionnaire. |
| --- |

**Section 1: Socio-demographic information**

Interview date (dd-mm-yy Buddhist Era.) _ _-_ _-_ _

1.1 Were you born male or female?

Male (1) _

Female (2) _

1.2 What was your age last birthday?

_ _ Years

1.3 What is your religion?

Buddhism (1) _ Christianity (2) _

Islam (3) _ No religion (4) _

Other (Specify) _______________ (5) _

1.4 Where did you mostly spend your life when you were young (aged less than 15 years)?

In the Thailand (1) _ Out of Thailand (specify) _______________ (2) _

1.5 What is the race/ethnicity of you? (You may choose more than one answer)

Chinese (1) _ Burmese (2) _

Shan (3) _ Hill tribes (specify) ____________ (4) _

Thai, another region (5) _ Other (specify) _______________ (6) _

1.6 What is your current living situation?

Live in my home (1) _ Live in rented room or house (2) _

Live in school dormitory (3) _ Other (specify) ___­­­________ (4) _

1.6.1 Who do you live with? (You may choose more than one answer)

Live with parent(s) / relative(s) (1) _

Live with employer(s) (2) _

Live with friend(s) (3) _

Live alone (4) _

Live with boyfriend/girlfriend or loved one (5) _

Other (specify) ___________ (6) _

1.7 Are you currently attending school?

Yes, at public or private school (1) _ Yes, at non-formal education center (2) _

No (3) _ (If no skip to Q 1.8) Other (specify) ___________ (4) _

1.7.1 What level of education are you attending?

Primary level (1) _

Secondary level (2) _

High-school level (3) _

1.8 Do you do work for which you receive income?

No (1) _

Yes (Specify)________________Bath/Month (1) _

**Section 2: Lifestyle information**

***In this section we would like to ask some questions about your lifestyle***

2.1 Do you currently have access at home to the following items?

|  | **I own one myself** | **I have access to one my family owns** | **No, I do not have access to one at home** |
| --- | --- | --- | --- |
| Mobile phone |  |  |  |
| Computer |  |  |  |
| Internet |  |  |  |
| Television |  |  |  |
| Motorcycle |  |  |  |
| Pick-up truck |  |  |  |
| Car |  |  |  |

2.2 Have you ever drunk alcohol (e.g. beer, whisky, wine) during the past year?

Yes (1) _

No (2) _

***If no skip to Q 2.3***

2.2.1 If yes, how often did you drink?

Less than once a week (1) _

About once a week (2) _

More than once a week (3) _

2.2.2 On average, how many drinks did you usually have at one time? (One drink = one beer, one whisky-soda, one glass of wine, etc.)

1-2 drinks (1) _

3-5 drinks (2) _

More than 5 drinks (3) _

2.3 Have you ever smoked cigarettes during the past year?

Yes (1) _

No (2) _

***If no skip to Q* 2.4**

2.3.1 If yes, on average, how many cigarettes per day have you smoked?

Less than 1 – I smoke occasionally (1) _

1-5 cigarettes (2) _

6-10 cigarettes (3) _

More than 10 cigarettes (4) _

2.4 During the past year, how often have you taken the following drugs?

|  | **Regularly*,**  **with approximately** | **Occasionally** | **Never** |
| --- | --- | --- | --- |
| Methamphetamines *(yaa baa)* | __ __ time(s)/month |  |  |
| Marijuana (*ganja*) | __ __ time(s)/month |  |  |
| Glue | __ __ time(s)/month |  |  |
| Sedatives | __ __ time(s)/month |  |  |
| Tranquilizers | __ __ time(s)/month |  |  |
| Ecstasy (*yaa e*) | __ __ time(s)/month |  |  |
| Ketamine (*yaa* k) | __ __ time(s)/month |  |  |
| Ice drugs (*yaa ice*) | __ __ time(s)/month |  |  |
| Heroin (non-injected) | __ __ time(s)/month |  |  |
| Injected any illegal drugs | __ __ time(s)/month |  |  |
| Other (specify) ________________ | __ __ time(s)/month |  |  |

* Regularly means you take at least once a month on that type of drug. **Section 3: Relationship information**

***Young people have various views about love and intimate relationships. Please read the following statements and indicate whether you agree or disagree with each of them.***

3.1 In the past year, who did you mainly spend your free time with? (You may give more than one answer)

Friends (1) _

Boyfriend/girlfriend (2) _

Mother (3) _

Father (4) _

Siblings (5) _

Other relatives (6) _

Myself, on my own (7) _

3.2 Do you currently have a boyfriend/girlfriend?

Yes (1) _

No (2) _

***If no, skip to Section 4***

3.2.1 How old is your boyfriend/girlfriend?

_ _ years

3.2.2 Are they male or female?

Male (1) _

Female (2) _

3.2.3 Do you plan to get married / have a permanent relationship with him / her?

Yes (1) _

No (2) _

Not sure (3) _

3.2.4 Have you had sex with your boyfriend/girlfriend?

Yes (1) _

No (2) _

3.2.5 Do you live with your boyfriend/girlfriend?

Yes (1) _

No (2) _

**Section 4: Sexual experience**

***The following questions are about your sexual experience***.

4.1 Have you ever had **sexual intercourse**?

***[Sexual intercourse*** *means that one person’s penis was inserted in the vagina or anus of another person.]*

Yes (1) _

No (2) _

***If never had sexual intercourse, skip to Section 7 (Reasons for not having sexual intercourse)***

4.2 How many different people have you ever had sexual contact/intercourse with?

__ __ Persons

4.3 How old were you when you had sexual contact/intercourse for the **first time**?

Age _ _ years old

4.3.1 Where did it happen?

My room/house (1) _ Partner’s room/house (2) _

Friend’s room/house (3) _ Motel or hotel (4) _

Car (5) _ Brothel (6) _

Outdoors (7) _ Other (specify) ___________ (8) _

4.3.2 Who was your first sex partner?

Boyfriend/girlfriend (1) _

Other friend (2) _

Relative (3) _

Neighbour (4) _

Stranger (5) _

Sex worker (6) _

Other (specify) _______________ (7) _

4.3.3 Was your partner a male or a female?

Male (1) _

Female (2) _

4.3.4 **The first time** you had sexual intercourse, which method did you and your partner

use to avoid diseases / pregnancy? (You may give more than one answer)

Withdrawal of penis before ejaculation (1) _

Condom (2) _

Morning after pill (3) _

Germ killers (tablets or gel) (4) _

Traditional herbal medicines (5) _

We did not use any method (6) _

Other (specify) _______________ (7) _

4.4 When was the **last time** you had sexual contact/intercourse?

Within the last two days (1) _

Within the last week (2) _

Within the last month (3) _

Within the last 3 months (4) _

Within the last year (5) _

More than a year ago (6) _

4.4.1 Who was your last sex partner?

Boyfriend/girlfriend (1) _

Other friend (2) _

Relative (3) _

Neighbour (4) _

Stranger (5) _

Sex worker (6) _

Other (specify) _______________ (7) _

4.4.2 Was your partner on that occasion a male or a female?

Male (1) _

Female (2) _

4.4.3 **The last time** you had sexual intercourse, which method did you and your partner

use to avoid diseases / pregnancy? (You may give more than one answer)

Withdrawal of penis before ejaculation (1) _

Condom (2) _

Oral pill (3) _

Morning after pill (4) _

Germ killers (tablets or gel) (5) _

Traditional herbal medicines (6) _

We did not use any method (7) _

Other (specify) _______________ (8) _

4.5 Have you ever been forced (either physically or psychologically) to have ***sexual contact/ intercourse against your will*?**

Yes (1) _

No (2) _

***If the answer is No, skip to section 5***

4.5.1 At what age did this happen for the first time?

Age _ _

4.5.2 Who was your sex partner on that occasion?

Boyfriend/girlfriend (1) _

Friend (2) _

Relative (3) _

Neighbour (4) _

Stranger (5) _

Other (specify) ____________ (6) _

***THIS SECTION ONLY FOR THOSE WHO HAVE EXPERIENCED SEXUAL INTERCOURSE***

***Those who have never experienced sexual intercourse please skip to Section 7***

**Section 5: Sexually transmitted diseases**

***This section asks about your concerns about sexually transmitted diseases***

5.1 Have you ever experienced any of following signs / symptoms / diseases?

|  | **Yes** | **No** | **Not sure / Don’t know** |
| --- | --- | --- | --- |
| Ulcer or sore in genital area |  |  |  |
| Painful/burning sensation when urinating |  |  |  |
| Itching around the opening of the penis/vagina |  |  |  |
| Abnormal genital discharge (a white, yellow, or green discharge from penis/vagina) |  |  |  |
| Vaginitis/Cervicitis (females only) |  |  |  |
| Pelvic Inflammatory Disease (females only) |  |  |  |
| Urethritis |  |  |  |
| Gonorrhoea |  |  |  |
| Syphilis |  |  |  |
| Chlamydia |  |  |  |
| Genital herpes |  |  |  |
| HIV infection |  |  |  |
| Other sexually transmitted disease (specify)___________ |  |  |  |

***If No or Not sure / Don’t know to ALL items, skip to Section 6***

5.1.1 If yes to any item listed, the last time you had one of the above symptoms, what did you do?

Visited doctor at public hospital/clinic (1) _

Visited doctor at private hospital/clinic (2) _

Went to pharmacy asking for advice and to buy drugs (3) _

Bought drugs from somewhere else to treat myself (4) _

Did nothing (5) _

Other (specify) _______________ (6) _

***THIS SECTION ONLY FOR THOSE WHO HAVE EXPERIENCED SEXUAL INTERCOURSE***

***Those who have never experienced sexual intercourse please skip to Section 7***

**Section 6: Birth control, pregnancy and abortion**

***This section asks about your worries concerning birth control, pregnancy and abortion***

6.1 Since you first had sexual intercourse, how regularly have you and your partner(s) used each of the following methods of birth control?

|  | **All of the time** | **Most of the time** | **Occasionally** | **Never** |
| --- | --- | --- | --- | --- |
| Withdrawal of penis before ejaculation |  |  |  |  |
| Periodic abstinence |  |  |  |  |
| Condom |  |  |  |  |
| Oral pill |  |  |  |  |
| Morning after pill |  |  |  |  |
| Injection |  |  |  |  |
| Intra-Uterine Device (IUD) |  |  |  |  |
| Norplant |  |  |  |  |
| Traditional herbal remedies |  |  |  |  |
| Other, specify___________ |  |  |  |  |

6.5 Have you ever been pregnant, or made a sexual partner of yours pregnant?

Yes (1) _

No (2) _

***If no, skip to Section 8***

6.5.1 If yes, how many times?

_ Time (s)

6.5.2 What were the outcomes of the pregnancies? (You may give more than one answer)

Abortion (1) _

Miscarriage (2) _

Delivery (3) _

Still pregnant (4) _

Other, specify___________ (5) _

***If you/your partner have never had an abortion, skip to Section* 8**

6.5.3 If abortion, how many times have you or sexual partners ever had an abortion?

_ Time (s)

6.5.4 How did you or your sexual partner go about seeking an abortion last time? (You may give more than one answer)

Went to a private clinic / hospital (1) _

Went to a public health organization (2) _

Visited traditional healer (3) _

Had friend help by buying abortifacients (4) _

Did it by myself/herself (5) _

Don’t know (6) _

Other (specify) _______________ (7) _

6.5.5 How much did you/your sexual partner pay for having that abortion?

Less than 1,000 Baht (1) _

1,000 – 2,499 Baht (2) _

2,500 – 4,999 Baht (3) _

5,000 Baht or more (4) _

6.5.6 What was the outcome of the most recent abortion?

No complications at all (1) _

Minor complications and I/she did nothing (2) _

Minor complications and I/she got some medicines from drug store (3) _

Severe complications and I/she went to clinic/hospital (4) _

Don’t know (5) _

Other (specify) _______________ (6) _

***When you complete this section, please skip to Section 8 THIS SECTION ONLY FOR THOSE WHO HAVE NEVER EXPERIENCED SEXUAL INTERCOURSE***

***Those who have experienced sexual intercourse please skip to Section 8.***

**Section 7: Reasons for not having sexual intercourse**

People may have mixed reasons for not having sexual intercourse. Please read the following statements and indicate whether you agree or disagree with each.

7.1 Reasons why you may not have had sexual intercourse:

|  | **Yes** | **No** | **Not sure** |
| --- | --- | --- | --- |
| I don't feel ready to have sex. |  |  |  |
| I have not had the opportunity to have sex. |  |  |  |
| I think that sex before marriage is wrong**.** |  |  |  |
| I am afraid of getting pregnant or causing a pregnancy if I have sex**.** |  |  |  |
| I am afraid of getting HIV/AIDS or another sexually transmitted infection if I have sex. |  |  |  |
| I don’t have sex because I care about my parents’ feelings. |  |  |  |
| I am afraid of negative reactions from school / workplace if I have sex. |  |  |  |
| I will feel badly about myself if I have sex before marriage. |  |  |  |
| Other (specify) __________________________ |  |  |  |

7.2 Which of these statements best describes your plans regarding having sex?

I plan to wait until marriage (1) _

I plan to wait until I am engaged to be married (2) _

I plan to wait until I find someone I love (3) _

I plan to have sexual intercourse whenever an opportunity comes along (4) _

I plan not to have sexual intercourse with anyone (5) _

I am not sure about whether or when I will have sex in the future (6) _

Other (specify) _______________ (7) _

7.3 Do you feel any pressure from others to have sexual intercourse?

A great deal (1) _

A little (2) _

None (3) _

***If none, skip to Section* 8**

7.3.1 From whom do you feel pressure? (You may answer more than one choice)

Boyfriend/girlfriend (1) _

Same sex friends (2) _

Friends of the opposite sex (3) _

Mass media (4) _

Other (specify) _______________ (5) _

**Section 8: Use and perceptions of sexual health services**

***In this section, we’d like to know about your experiences and perceptions regarding sexual health services***

8.1 Have you ever visited a health facility or doctor of any kind to receive services or information on contraception, pregnancy, abortion or sexually transmitted dieases?

Yes (1) _

No (2) _ ***If no, skip to Q 8.7***

8.2 If yes, how many times have you sought services or information of that type from health personnel?

__ __ times

8.3 When you last saw a doctor or a nurse for that type of service, what was your reason for going?

STD (1) _

Contraceptives (2) _

Pregnancy test (3) _

Pregnancy termination (4) _

Maternal and child health (5) _

Other (specify) _______________ (6) _

8.4 Thinking about that last visit, did you go to a government or private health organization, or a centre at an NGO?

Private (1) _

Government (2) _

NGO (3) _

Other (specify) _______________ (4) _

8.5 At that time were you satisfied with the services provided by the health personnel?

Yes (1) _

No (2) _

8.6 Was there enough confidentiality?

Yes (1) _

No (2) _

Not sure (3) _

8.7 Do you think there should be a sexual health clinic which provides services only for young people?

Yes (1) _

No (2) _

Not sure / need more information (3) _

**Section 9: Experience of Testing for HIV/AIDS**

| No | Questions | Answer options | | | | | |
| --- | --- | --- | --- | --- | --- | --- | --- |
| 9.1 | Have you ever been tested for HIV/AIDS? | Yes | No |  |  |  | |
|  |  | 1 | 2 |  |  |  | |
| 9.2 | Have you tested for HIV in the last 12 months? | Yes | No |  |  |  | |
|  |  | 1 | 2 |  |  |  | |
| 9.3 | It is very likely that i will get tested in the next coming | Very unlikely | Somewhat unlikely | Somewhat likely | Very likely | Not sure | |
|  |  | 1 | 2 | 3 | 4 | 5 | |
| 9.4 | Getting tested for HIV is a responsible thing to do | Yes | No |  |  |  | |
|  |  | 1 | 2 |  |  |  | |
| 9.5 | How difficult do you think it is to find a location nearby to get tested for HIV? | Very difficult | Difficult | Easy | Very easy | Not sure | |
|  |  | 1 | 2 | 3 | 4 | 5 | |
| 9.6 | I fear the results of the HIV test |  |  |  |  | | |
|  |  | 1 | 2 | 3 |  | | |
| 9.7 | I think I am able to go and get tested for a HIV test | Yes | No | Not sure |  | |  |
|  |  | 1 | 2 | 3 |  | |  |
| 9.8 | I think I am able to go and get tested for a HIV test even if I fear the results | Yes | No | Not sure |  | |  |
|  |  | 1 | 2 | 3 |  | |  |
| 9.9 | My family (parents, siblings) find it important I have myself tested for HIV frequently | Yes | No | Not sure |  | |  |
|  |  | 1 | 2 | 3 |  | |  |
| 9.10 | My partner(s) find it important I have myself tested for HIV frequently | Yes | No | Not sure |  | |  |
|  |  | 1 | 2 | 3 |  | |  |
| 9.11 | My friends find it important I have myself tested for HIV frequently | Yes | No | Not sure |  | |  |
|  |  | 1 | 2 | 3 |  | |  |
| 9.12 | Do you think that you are “now” at risk of HIV? | High risk | Moderate risk | Low risk | No risk | | Not sure |
|  |  | 1 | 2 | 3 | 4 | | 5 |
| 9.13 | Do you think that you are “now” at risk of STI? | High risk | Moderate risk | Low risk | No risk | | Not sure |
|  |  | 1 | 2 | 3 | 4 | | 5 |
| 9.14 | What do think are barriers to HIV testing for young people like you? |  | | | | | |
| 9.15 | Which of the following methods would you prefer for HIV test? | Family based HIV testing at home | Voluntary HIV testing at health facility | HIV self-testing at home | Voluntary HIV testing in mobile clinic in the community | |  |
|  |  | 1 | 2 | 3 | 4 | |  |

***Thank you very much for taking time to answer the questionnaire.***
